# Laser Rangefinder Methods: Autonomous-Vehicle Trajectory Control in Horticultural Plantings

**DOI:** 10.3390/s24030982

**Published:** 2024-02-02

**Authors:** Alexey I. Kutyrev, Nikolay A. Kiktev, Igor G. Smirnov

**Affiliations:** 1 Department of Technologies and Machines for Horticulture, Viticulture and Nursery, Federal Scientific Agroengineering Center VIM, 1-st Institutsky Proezd, 5, 109428 Moscow, Russia; alexeykutyrev@gmail.com (A.I.K.); rashn-smirnov@yandex.ru (I.G.S.); 2 Department of Intelligent Technologies, Taras Shevchenko National University of Kyiv, Volodymyrs’ka Str., 64/13, 01601 Kyiv, Ukraine; 3 Department of Automation and Robotic Systems, National University of Life and Environmental Sciences of Ukraine, Heroiv Oborony Str., 03041 Kyiv, Ukraine

**Keywords:** LiDAR, bypass algorithm, robotic platform, positioning, point cloud, motion trajectory, application, industrial horticulture

## Abstract

This article presents a developed motion control system for a robotic platform based on laser-ranging methods, a graph traversal algorithm and the search for the optimal path. The algorithm was implemented in an agricultural building and in the field. As a result, the most efficient algorithm for finding the optimal path (A*) for the robotic platform was chosen when performing various technological operations. In the Rviz visualization environment, a program code was developed for planning the movement path and setting the points of the movement trajectory in real time. To find the optimal navigation graph in an artificial garden, an application was developed using the C# programming language and Visual Studio 2019. The results of the experiments showed that field conditions can differ significantly from laboratory conditions, while the positioning accuracy is significantly lower. The statistical processing of the experimental data showed that, for the movement of a robotic platform along a given trajectory in the field, the most effective conditions are as follows: speed: 2.5 km/h; illumination: 109,600 lux; distance to the tree: 0.5 m. An analysis of the operating parameters of the LiDAR sensor showed that it provides a high degree of positioning accuracy under various lighting conditions at various speeds in the aisles of a garden 3 m wide with an inter-stem distance of 1.5 m and a tree crown width of 0.5 m. The use of sensors—rangefinders of the optical range—allows for the performance of positional movements of the robotic platform and ensures the autonomous performance of the basic technological operations of the units in intensive gardens with a deviation from the specified trajectory of no more than 8.4 cm, which meets the agrotechnical requirements.

## 1. Introduction

The development of a motion control system for a robotic platform in agriculture, and particularly industrial horticulture [1], is due to several factors. The industry is labor-intensive, and efficiency and precision in resource management are key. The robot motion control system can solve a number of pressing problems in agriculture:

- It increases labor productivity, optimizes the use of resources, reduces fuel costs and increases the accuracy of operations;
- It reduces the use of chemical fertilizers and pesticides, providing the opportunity for more precise and targeted effects in soil and plants;
- It collects extensive data on the soil, plants and growing conditions in real time.

Such innovations improve production processes, contribute to preserving the environment and ensure food security. Monitoring plant health and the early detection of diseases or pests in gardens allows for taking timely measures to prevent them. These data can be used to more accurately predict crop yields, plan agricultural operations and optimize production processes. The optimized routing of robotic platforms can help reduce soil erosion, reducing the negative impact on the environment.

Navigating autonomous vehicles, including guided tractors and robotic platforms, in fields and industrial gardens is a complex task that requires additional research. The motion control algorithm of an autonomous vehicle is designed to build a route, avoid obstacles, stop the vehicle to perform operations (picking fruit, pruning trees, spraying, removing weeds, monitoring the garden, etc.), process and analyze information from sensors and determine a location at its coordinates [1,2,3].

Various navigation systems are known, but they have a number of disadvantages that are associated with the substantial technical requirements (RAM and external memory) and insufficient accuracy in determining the location of an autonomous vehicle to perform various technological operations [4].

To operate autonomous vehicles in garden plantings, control system developers distinguish between global, local and personal navigation [5,6]. The global navigation of an autonomous vehicle ensures route planning and movement along rows of plantings along the entire perimeter of an industrial garden. Local navigation provides the determination of the current coordinates of an autonomous vehicle and its movement in a limited area (for example, between trees). Personal navigation ensures the construction of a movement route during close interactions with objects (branches, fruits, weeds, etc.); that is, it determines the trajectory of the robotic device’s manipulator [7].

When determining the location and route of movement of an autonomous vehicle in an industrial garden, two types of methods for obtaining information can be used: passive (from global satellite systems (GLONASS/GPS), radio transmitters) and active (using its own sensors, laser rangefinders, stereo cameras, inertial navigation systems, etc.) [8,9,10,11]. Various types of sensors are used to detect trees, fruits, leaves, weeds and other obstacles in garden rows. Like methods of obtaining information, sensors can be passive or active. In industrial gardens, various types of cameras are used to determine the locations of autonomous vehicles: mono- and stereo cameras that determine the distance to the harvested fruit or weed, RGB cameras (which determine the color and shape of the fruit or leaves, as well as signs of their diseases, in 2D format with high resolution), thermal imaging and hyperspectral cameras [12,13]. Cameras are classified as passive sensors.

**Figure 1:**
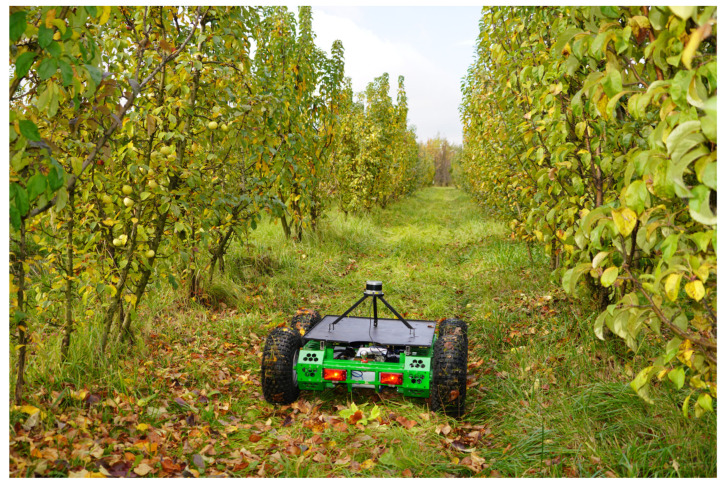
Conducting a field experiment.

Active sensors are distinguished by the fact that they send impulses to objects and receive responses from them. In an industrial garden, signals such as laser (clouds of points, 3D matrices and digital twins), ultrasonic and radio signals are used to determine the location and movement of an autonomous vehicle [14,15]. Speed, acceleration and changes in the position of the grippers and other working parts when the manipulator is operating under field conditions are controlled using accelerometers, gyroscopes and global satellite systems [16,17]. In this study, we will consider the use of a LiDAR (Light Detection and Ranging) sensor to construct maps of the obstacles and trajectories of an autonomous vehicle under the field conditions of an industrial garden. These sensors are most suitable for this task, as they have a 360-degree field of view, detect obstacles in the form of trees and other vehicles and construct maps with fairly high accuracy (up to several centimeters) (Figure 1). In this case, big data are generated based on points or pixels, the processing of which is carried out using cluster analysis methods, the location is determined and the trajectory of the further movement of the autonomous vehicle is constructed.

A comparative analysis of various types of sensors used for the navigation of robotic platforms is given by the authors of [18,19,20,21] and shows that LiDAR sensors have an advantage over other sensors and cameras for the navigation of robotic systems in horticulture. Analyses of existing control systems for autonomous vehicles and studies conducted in different countries [22,23,24,25] have shown that the following types of algorithms can be used for navigation and control in industrial gardens: the hybrid-navigation, Dijkstra, potential-field, SLAM and vector pursuit algorithms, among others [18]. An analysis of the work of well-known algorithms [18] showed that the use of the A* algorithm to traverse the graph and find the optimal path makes it possible to determine, with a high degree of accuracy, the location of an autonomous vehicle in field conditions when it moves between rows and moves to another row to collect fruits, monitor trees, prune, spray and perform other tasks [18].

The most modern tool used for making decisions on process management in agronomy and horticulture is machine learning. The relevance of its application is due to the complexity of the task of analyzing plantings and predicting the possible harvest, as the overall results are influenced by many factors. This tool is also successfully used to analyze information obtained from monitoring the state of the environment and for identifying diseases of leaves and fruits, their ripeness, weeds, etc. [26,27,28].

## 2. Literature Review

Various scientists of the world are engaged in research on and improvements in navigation systems. In an article by the Chinese researchers Lu Lu et al. (2023) [29] from Chongqing Jiaotong University, the authors propose a rather interesting solution regarding 3D reconstruction that merges monocular vision and LiDAR. The authors consider this method in the context of urban development and vehicle navigation along city streets.

The researchers Chen, P.-Y. et al. (2022) [30] from the Ching-Yi National University of Technology (Tai-wan) proposed a method for determining the distance to an object by using 3D LiDAR and merging heterogeneous sensors with a camera. This development is interesting in that the surveillance system is installed on a moving vehicle, and the Yolo-4 neural network is used to recognize objects.

The use of LiDAR sensors in conjunction with multispectral imaging (MSI) for the classification of tree species was proposed by the researchers Li, Q. et al. (2023) from Canada and China [31]. This method made it possible to improve the accuracy of classifying trees in the forest compared to using separate recognition tools.

The researchers Liu, Y. et al. [32] from the Guilin University of Technology (China) proposed a method for tree crown recognition that fuses LiDAR data and high-resolution stereo images, which were collected using a UAV. At the same time, the PointNet ++ neural network was used for recognition. The results of the data fusion showed an improvement in the segmentation of the image of tree crowns compared to using them separately.

The classification of tree species using the LiDAR sensor was carried out by the researchers Z. Hui et al. (2023) from the East China University of Technology [33]. The method proposed by them lies in the fact that the feature vectors were specially developed based on fractal geometry, including the fractal dimension and intersection point. This made it possible to improve the classification of trees. A number of researchers have been engaged in the adaptation of and improvement in navigation aids in horticulture. The U.S. and Brazilian researchers M. G. Raman et al. (2022) [34] recognized high-resolution RGB images in a peach orchard obtained in various nadir and oblique images. The authors processed and created datasets of orthomosaics and DSM, DTM, 3D UAV and LiDAR point clouds to measure the peach tree height and crown volume.

The Australian researchers P. Moghadam et al. (2022) [35] proposed the use of a digital twin to display the state of the horticulture in a 3D model. In the article, the authors present an automated system for the dynamic monitoring of tree crowns to create a digital twin of each tree in a large horticultural field. The AgScan3D+ system consists of rotating 3D LiDAR and cameras that can be mounted on an agricultural vehicle and that provide real-time decision support to the farm by monitoring the status of each plant in 3D, such as its health, structure, stress, fruit quality and more.

The researchers Wu, D. et al. (2018) [36] from Australia and Saudi Arabia estimated the following parameters using ground-based laser scanning based on LiDAR sensors: changes in the leaf areas, the leaf area densities and the vertical profiles of the leaf areas for the crowns of mango, avocado and macadamia trees. The Korean researchers T.T.H. Giang et al. (2023) [37] proposed a method for detecting the cut point of sweet pepper leaves in 3D space using 3D point clouds. The robot’s arms move to the recognized positions and cut the leaves. The result is achieved using semantic segmentation neural networks, the ICP algorithm and ORB-SLAM3, and the SLAM visual application with the LiDAR camera. This 3D point cloud is made up of plant parts that have been recognized by the neural network.

Quite close to the topic of our article is the work of the Chinese researchers Tang, J. et al. (2022) [38]. The authors determined the yield of butter tea (*Camellia oleifera*) by using color spaces to identify the point clouds of oily fruits. In this study, an optimized mean-shift-clustering algorithm was used to improve the efficiency and accuracy of the crop identification. It was created to extract the tea-tree-oil point cloud and identify the product. Tea-tree-oil point cloud data were obtained using ground laser scanning. The work of the researchers J. Iqbal et al. (2020) [39] from the University of Georgia (the U.S.) describes the modeling of an autonomous mobile robot for phenotyping and navigation in the field based on LiDAR, carried out by the authors. The authors present a mobile field robot based on the Robotic Operating System (ROS), which simultaneously moves through closed rows of crops and performs various phenotyping tasks, such as measuring the plant volume and canopy height. The researchers created a highly accurate model of a cotton plant in Sketchup TM and imported it into Gazebo as a 3D graphic file. After creating a single-plant model, the plants were randomly rotated and grouped together in Sketchup TM to create plots. According to the authors, the application of a hybrid strategy with GPS waypoint tracking and LiDAR-based navigation moved the robot through an agricultural field with an RMS error of 0.0778 m, which was 0.2% of the total distance traveled.

Laboratory and field tests of the height recognition of cotton plants using LiDAR are described in an article by the American researchers Sun, S. et al. [40]. The authors of the study used a tractor with a sprayer as a data collection platform. The LiDAR sensor unit was attached to the sensor bar at the rear of the tractor. An RTK-GPS device was used to provide spatial LiDAR coordinates at all times. The data collected with the LiDAR were recorded by a rugged laptop computer via an Ethernet interface. A dense 3D model of the cotton plants was obtained by moving the tractor along the rows of the field. In the described study, the height of a cotton plant was determined (maximum height: −1824 mm), while the LiDAR simultaneously scanned three rows of plants in the field from above. H. Moreno et al. (2020) [41] describe the reconstruction of a vineyard on the ground using an automated system based on LiDAR. The 3D map was matched to the ground truth, which was manually determined by trimming off the remaining weight. The number of LiDAR scans affected the relationship with the actual biomass measurements and had a significant effect on the treatment.

Researchers from Ukraine implemented an algorithm for moving a mobile robot in the space of a greenhouse [42]. To perform this, the robot must pass certain checkpoints along its path, set by the operator before starting. If there is an obstacle on the route, then the robot detects it with an ultrasonic sensor; if it is possible to bypass the obstacle, then it maneuvers; if it is impossible to reach a certain destination, then the robot gives a sound signal, sends a message to the operator and moves on to the next destination. To simplify the orientation, the space of the greenhouse was conditionally divided into sectors; the robot kept track of the sector change using the colors of the labels on the plant pots. To build the information system of a mobile robot, a free and open software shell ROS (Robot Operating System, Stable release Iron Irwini/23 May 2023) was used. The disadvantages of the development include the fact that the described robot is on rails, which was suitable for the greenhouse and premises but is not suitable for horticulture. The researchers D. Komarchuk et al. [43,44] describe drone navigation in an industrial greenhouse. The authors solved the problem of planning the UAV flight trajectory in a complex conflict situation. To synthesize the optimal UAV trajectory in a greenhouse and in the field, the dynamic programming method was used with a generalized optimality criterion according to a nonlinear compromise scheme.

Research by Chinese scientists [45,46,47,48] examines algorithms for the construction of ship trajectories. An adaptive control algorithm under conditions of uncertainty using the backward-step method provides the optimal tracking of the vessel using neural networks [45]. The study [46] presents an optimized formation control algorithm for unmanned surface vessels taking into account collision avoidance using radial basis neural networks to model uncertainties. The papers [47,48] solve the problem of observer tracking and convergence in optimal control using adaptive/approximate reinforcement learning, which allows the entire surface vessel system to be considered with a single dynamic equation. An analysis of these studies showed that the reinforcement learning methods used, especially those using neural networks, are complex and require large computational resources. This may result in slower control processes when used in real time. Training methods require extensive and varied training data. Insufficient or inadequate data can lead to incorrect learning and unsatisfactory results.

To solve the problem, we propose a laser method, a graph algorithm and a search for the optimal movement path for the robot in agriculture. However, depending on the environmental conditions, the laser sensor may experience limitations in visibility, which may also reduce its effectiveness. The proposed approach can be implemented both in the laboratory and in the field, which provides a wider range of applications. The experimental results highlight the importance of adapting algorithms to field conditions and achieving a high positioning accuracy in orchard conditions.

Under the modern conditions of horticulture, the environmental factor is of significant importance, and the solution to the problem of long-term planning according to the Leontiev–Ford ecological and economic model, taking into account the magnitude of the environmental costs, is proposed by the authors Gnatienko G.N. et al. in [49].

According to the results of the literature review, it can be stated that navigation using the LiDAR sensor is widely used in agriculture, and particularly in agronomy. However, to date, few studies have been carried out in the orchards of the Central European zone, particularly when monitoring apple orchards. Also, a number of applied problems of navigation in horticulture remain unresolved, including the following:

- The selection of the optimal algorithm for the movement of the robotic platform in the horticultural environment;
- The building of an optimal route of movement in the horticultural environment;
- The study of the dependence of the navigation accuracy on various factors: the illumination, platform speed and distance to the tree;
- Finding the optimal values of these factors.

Thus, the purposes of this study were to select the optimal trajectory of the robotic platform in rows of horticulture plantings based on laser-ranging methods to select the best algorithm for bypassing the graph and finding the optimal path, and to substantiate the factors to ensure a high positioning accuracy when performing various technological operations.

## 3. Materials and Methods

### 3.1. Description of Robotic Platform with LiDAR Sensor

To test the laser-ranging method indoors with artificial trees and in the field conditions of an industrial orchard, a small autonomous vehicle developed by the authors was used, on which a Velodyne VLP-16 LiDAR sensor was installed (produced by Velodyne Lidar, San Jose, CA, USA). The main parameters of this sensor are as follows: laser wavelength: 905 nm; scanning frequency: 300 kHz: maximum field-of-view range: 360°; mirror rotation speed: 20 rpm.

**Figure 2:**
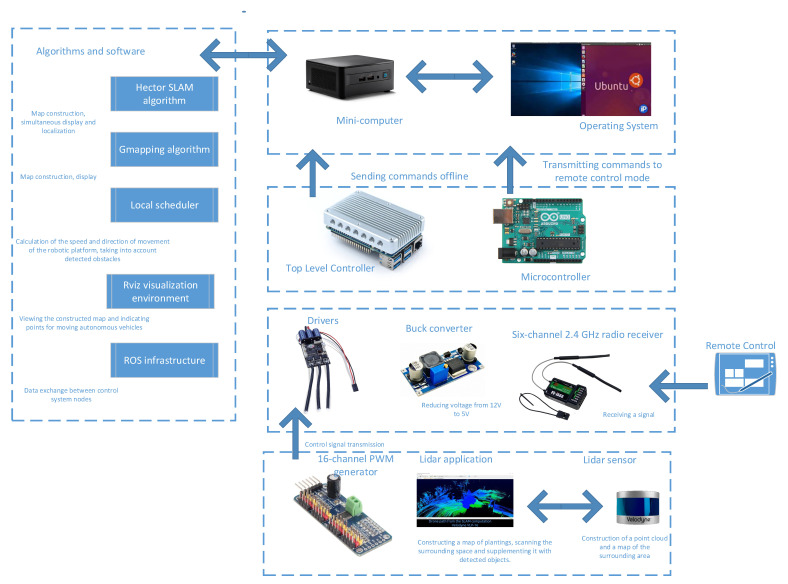
Architecture of an autonomous-vehicle navigation control system based on the use of a LiDAR sensor.

The autonomous platform is controlled by a multi-level and multi-tasking hardware–software complex, the architecture of which is shown in Figure 2.

The control structure can be expanded and adapted for various mobile robotic devices, such as surface vessels, quadcopters and others. Each type of mobile robot may have unique characteristics and requirements, so expanding the control structure must take these features into account. The proposed control structure is differentiated to adapt to various conditions in the aquatic environment and in three-dimensional airspace, and it provides for the optimization of the energy consumption to increase the autonomy of various mobile robotic devices. Considering the variety of mobile robots, the structure includes communication systems that ensure efficient data exchange between the robot and operator, as well as between different robots if collective control is used. For mobile robots operating in different environments, such as surface vessels or quadcopters, it is important to incorporate obstacle detection and avoidance algorithms into the control structure, taking into account the specific environmental conditions.

### 3.2. Finding a Path in a Graph with Obstacles

An example of finding a path in a graph with obstacles (1–10) using the various algorithms of the Github PathFindings web service is shown in our previous work [18]. Based on the results of finding a path in a graph with obstacles by comparing four algorithms in the Github PathFindings web service, we obtained the data presented in Table 1. To summarize, we can say that, for our multi-purpose field robotic platform for agricultural purposes and, in this case, for working in an apple orchard, the optimal path-length algorithm is the breadth-first search algorithm, although it is inferior in terms of the number of operations. Algorithm A* is the best in terms of the time criterion.

### 3.3. Modeling the Optimal Robot Route

#### 3.3.1. Formulation of the Problem

It is necessary to create a software application that implements a movement algorithm in agricultural buildings or gardens. At the initial stage, the robot is already in the room. It must first analyze the space and find an unoccupied area. The room and occupied area can be arbitrary. The user has the opportunity to enter a point towards which the robot should move, while following the most optimal path. The user can also stop the work or select another action that the robot can perform. The robot completes its work when it reaches the end point.

#### 3.3.2. Movement Algorithm Work in Agricultural Premises

Because it is important for us that the robot chooses the shortest path, we propose to divide the robot’s algorithm into two phases, namely, the learning phase and the search phase. In the training phase, we generate n graphs on the 2D plane and store only those that do not border or are in obstacles. The next step is to connect the nodes using the k-nearest-neighbor (kNN) method. The essence of the method is to find k objects that are closest to a certain point, and the relationship of this point to those objects that are closest:

f(n) = g(n) + h(n)(1)

where g(n) is the “cost of the path” from the starting point (n) to an arbitrary final point in the garden, h(n) is the ranking function of the alternative path and f(n) is the minimum “cost” of the transition to the neighboring point of the trajectory.

At each iteration of the pathfinding algorithm, the minimum “cost” [50] is calculated (that is, moving to the next tree or stopping point of the autonomous vehicle).

#### 3.3.3. Implementation of the Movement Algorithm Work in Agricultural Premises

The following classes are used to implement the robot movement algorithm in agricultural premises [50]:

(1) The structure “Node” is responsible for saving the parameters of the nodes;
(2) The “Obstacle” class is intended to describe the position of an obstacle in a room. It contains a point that defines where the obstacle starts from and its height and width;
(3) The “Path” structure is responsible for the two closest nodes between each other. It contains two “Node” objects describing the locations of the nodes, as well as the transportation cost (in our case, the cost is the distance);
(4) The “Algorithm A*” class is used to find the optimal path for a given graph. It contains information about all the possible ways and their costs;
(5) The “Robot” class is the main class responsible for creating nodes, combining them into graphs, finding the optimal path and controlling the movement of the robot.

Thus, the user is able to create their own premises and obstacles. Also, the user can control the robot and indicate whether the robot should move or stand still.

### 3.4. 3D Visualization of Planning Path of Movement and Setting Points of Trajectory of Movement, and Methodology for Conducting Laboratory and Field Studies

**Figure 3:**
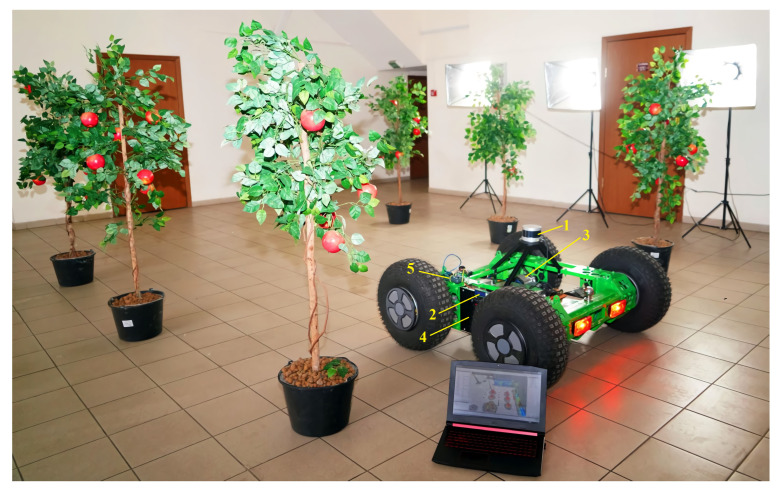
Conducting an experiment in a laboratory based on a robotic platform with a motion control system based on laser location methods: 1—LiDAR Velodyne Puck (VLP-16); 2—top-level control system unit; 3—BM1418ZXF DC motor; 4—Benewake TFmini Plus rangefinder; 5—system unit lower-level controls.

The best-path planning was carried out by the authors using the Rviz visualization environment and the Python programming language. The implemented A* algorithm processes the data obtained as a result of scanning obstacles with a LiDAR sensor and estimates the distance to the target. Next, experiments were carried out under laboratory conditions (in an enclosed room with artificial trees and artificial lighting) and under the field conditions of an industrial apple orchard. The purposes of the experiments were to evaluate the accuracy of determining the distance to an object by a LiDAR sensor installed on an autonomous vehicle, and to evaluate the development of the pathfinding algorithm in real conditions. The experiment in the rows of the artificial garden model is shown in Figure 3. The LiDAR sensor scans the surrounding space and builds a 3D cloud of location points in the X, Y and Z coordinates of the autonomous vehicle. Next, its location is determined, and at each iteration of the algorithm, the map is updated, the control strategy is determined and the control actions are transmitted to the controllers to set the vehicle in motion.

An analysis of the deviation in the real path of an autonomous vehicle from the specified initial, intermediate and final trajectory points, set at a distance of 0.5–1.5 m, was carried out. The illumination level varied from 10 to 110 thousand lux in increments of 50 thousand. The equipment for conducting the experiment under laboratory conditions is given in Table 2.

**Figure 4:**
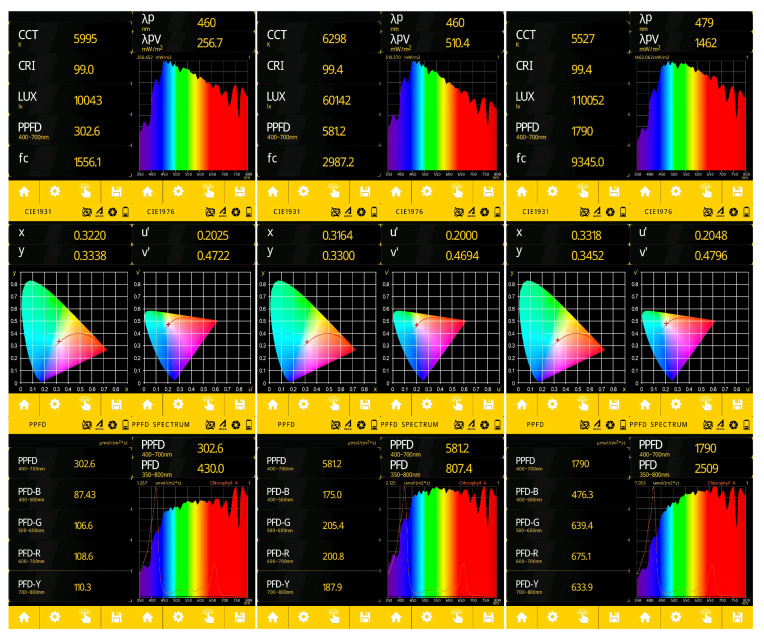
Illumination control during laboratory experiment (illumination: 10,000 lux, 60,000 lux, 110,000 lux).

**Figure 5:**
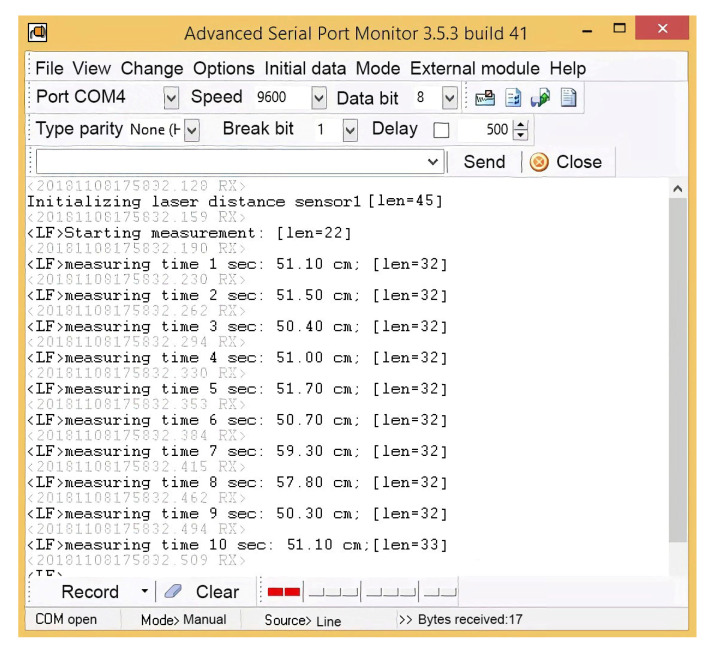
Monitoring of the COM port when the robotic platform moves along a given trajectory.

**Figure 6:**
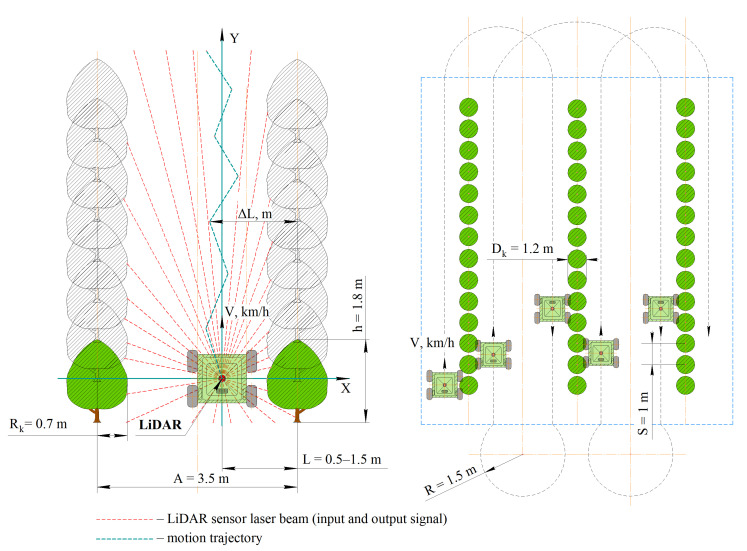
Parameters of the experimental site in the field (the method of the movement of the robotic platform is one-way shuttle).

The light control during a laboratory experiment using an Uprtek MF250N spectrometer is shown in Figure 4. The monitoring of the COM port while the robotic platform moves along a given trajectory using the Advanced Serial Port Monitor 3.5.3 program is shown in Figure 5. A field experiment was carried out using a similar technique. A plan of the apple orchard area where the navigation experiments were carried out is shown in Figure 6. The diagram shows an example of the dynamics of the robotic platform and input and output control signals.

## 4. Results

### 4.1. Implementation of the Search for the Optimal Path of the Robot in an Agricultural Building

When developing the program, the Windows Forms interface was used, as it provides the ability to implement a graphical interface faster and more efficiently (Pohorilyi D., 2023) [50]. The C# programming language was used to implement the project, and the development itself took place in Visual Studio 2019.

**Figure 7:**
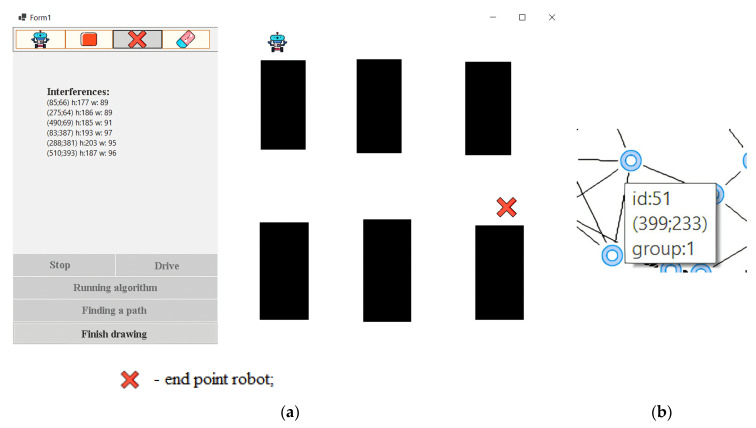
The initial position of the robot (**a**), and the window when hovering over the node (**b**).

**Figure 8:**
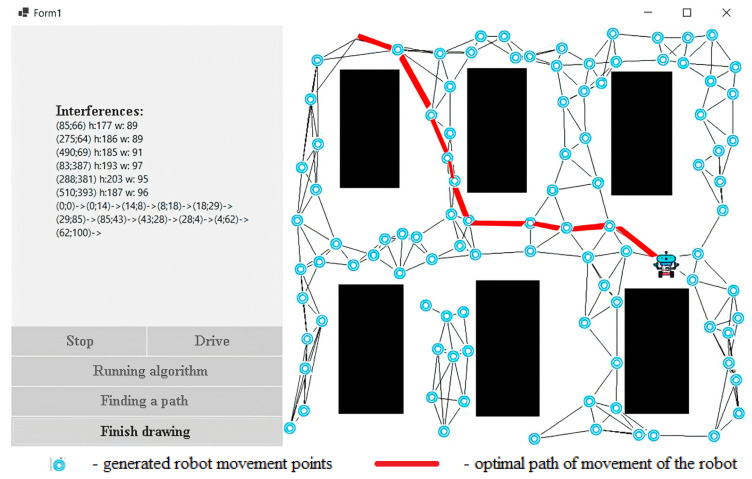
End-position work.

When the program [50] starts, the user is presented with an image that shows exactly where the interference is. Next, the user needs to draw a map, namely, the starting position of the robot, its end point and the obstacles. To perform this, the user clicks the Draw button. After clicking, a window is displayed (Figure 7). If the user has already drawn a map and wants to move on to the learning phase, they need to click the Finish Drawing button. Because the robot has not yet found the optimal path, it is more expedient for the user to press the “Search path” button. After that, 100 points will be generated, which will be connected to each other using the k-nearest-neighbor method. When the user hovers over a specific node, a window will appear that will display basic information about it (Figure 8).

**Figure 9:**
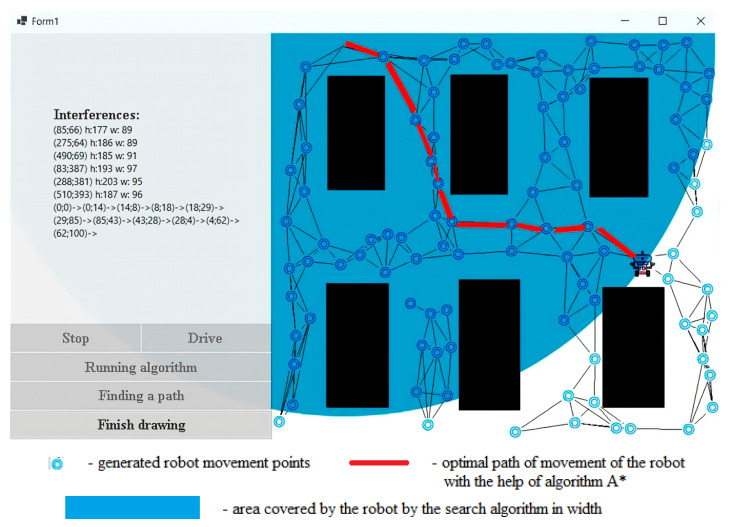
Breadth-first path search algorithm.

When using the breadth-first path search algorithm, we checked 77 nodes, which is 7.7 times more than that of our algorithm (Table 3). We see that the robot reached the end point only after traveling 10 nodes. The effectiveness of this algorithm in relation to the breadth-first search algorithm can be seen in Figure 9. 

### 4.2. Result of Constructing Trajectory of Robot Movement Based on Measurements of LiDAR Sensor

**Figure 10:**
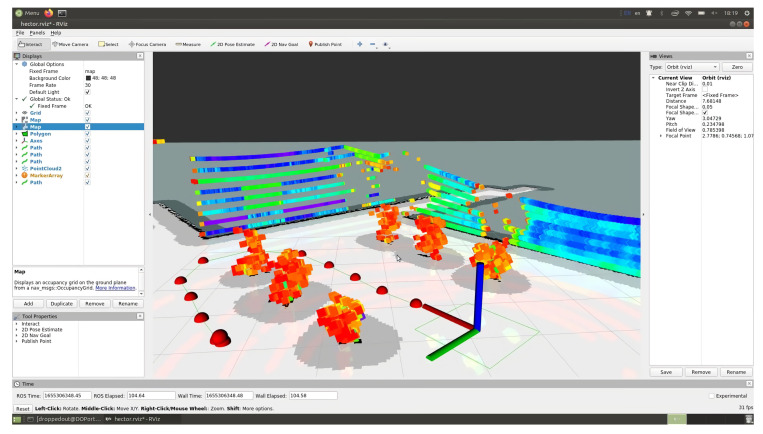
Visualization of the route by points using Rviz software in a room with artificial trees and lighting.

The route of a self-driving vehicle in an artificial garden was built using Rviz 1.14.20-1 software by specifying individual trajectory points on a map built using a LiDAR sensor. In Figure 10, the autonomous vehicle is marked with a green rectangular outline, with obstacles and the “danger zone” around it (detected using the LiDAR sensor) shown as a gray area. Experiments indoors with artificial trees were carried out three times, resulting in the determination of the maximum and minimum deviations from the given distance between the vehicle and the trajectory points (tree trunks).

**Figure 11:**
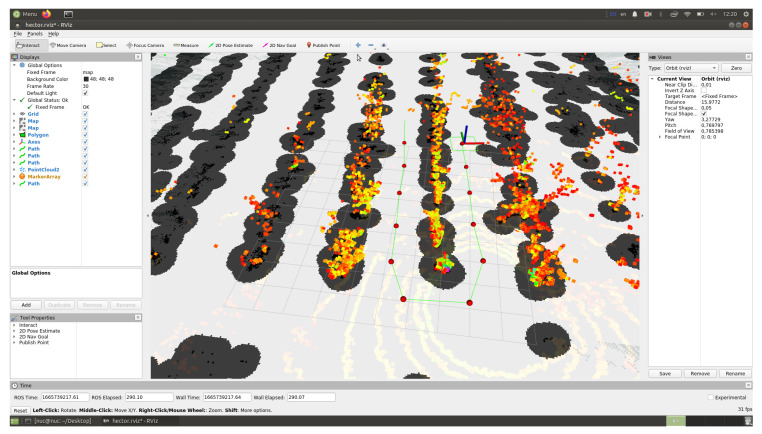
Building a motion trajectory by points in the Rviz visualization environment in the field.

The route of an autonomous vehicle moving across a field in the Rviz visualization environment was built by placing the starting, intermediate and ending points of the trajectory on a map of the surrounding area built using a LiDAR sensor (Figure 11).

### 4.3. Planning and Results of Experiment under Laboratory Conditions

**Figure 12:**
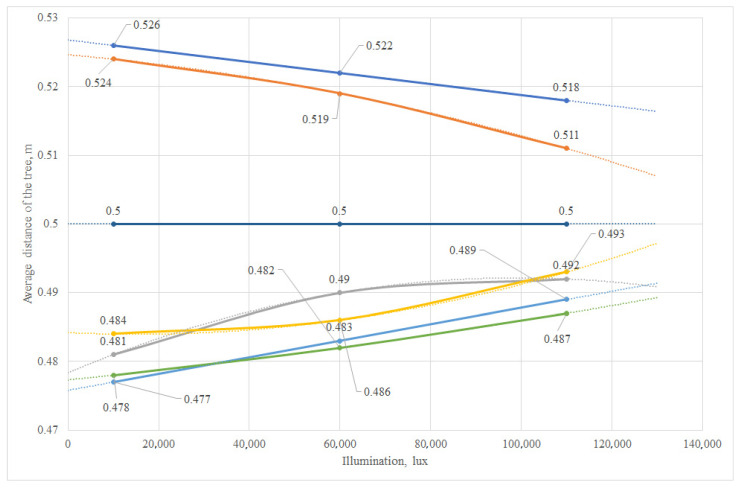
Graph of the deviation in the trajectory of the robotic platform at a speed of 2.5 km/h and the required (given) distance to the tree of 0.5 m.

**Figure 13:**
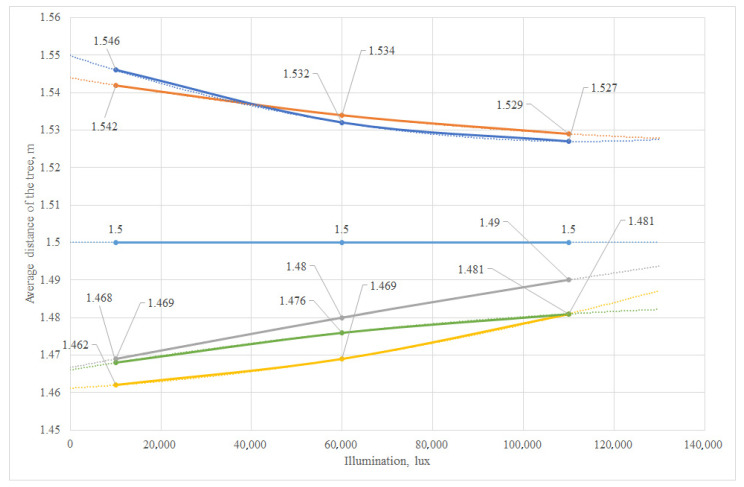
Graph of the deviation in the trajectory of the robotic platform at a speed of 3 km/h and the required (given) distance to the tree of 1.5 m.

As a result of the research, the range of variations, average linear deviation and standard deviation were determined, and graphs of the deviations in the vehicle trajectory from the illumination level at various specified distances to the tree from 0.5 to 1.5 m and a movement speed of 2.5–3.5 km/h were constructed (Figure 12 and Figure 13). The graphs for the six trees participating in the experiment are highlighted in different colors. The output parameter is the deviation value of the distance to the tree from the required one. The plan of the factorial experiment in an artificial garden is shown in [51], according to the methodology [52,53]. The purpose of the experiment was to determine the most efficient driving mode for an autonomous vehicle. The main significant factors when moving along a given trajectory are the speed of the vehicle, the illumination and the distance to the tree trunk.

The planning matrix, results of the experiment and values of the output coefficient are given in [51]. The linear interpolation was determined, and the variance in the reproducibility in parallel experiments was found. The results of the statistical data processing are shown in [51]. The coefficients of the mathematical model were found. The equation of the mathematical model was obtained in coded (2) and natural (3) forms:

y = 34.794 + 4.624 · x1 + (−3.041) · x2 + 3.041 · x3 + 0.959 · x1∙x2 + (−0.459) · x1∙x3 + 0.376 · x2∙x3 +(−1.623) · x1∙x2∙x3(2)

y = 29.398 + 0.988 · x1 + (−11.794) · x3 + 5.959 · x1 · x3(3)

The analysis of the mathematical model according to the Fisher criterion made it possible to determine the adequacy of the model ((F_p_(1.23) < F_t_(4.49)). As a result of the transformation, three variants of the mathematical model were obtained at y = f(x2, x3) at x1 = const (level 0; natural value: 3 km/h), y = f (x1, x3) at x2 = const (level 0; natural value: 60,000 lux) and y = f (x1, x2) with x_3_ = const (level 0; natural value: 1 m). Mathematical models taking into account the constant factor take the following forms:

y = 649.143 + (−59.072) · x2 + (2.516) · x3 + (−68.683) · x2^2^ + (−8.431) · x3^2^ + (10.877) · x2 · x3 (4)

y = 26.817 + 0.988 · x + (−12.698) · y +10.10 · x + 24.30 · y + (−1.841) · x · y(5)

y = 17.603 + 0.988 · x + 5.959 · x(6)

**Figure A1:**
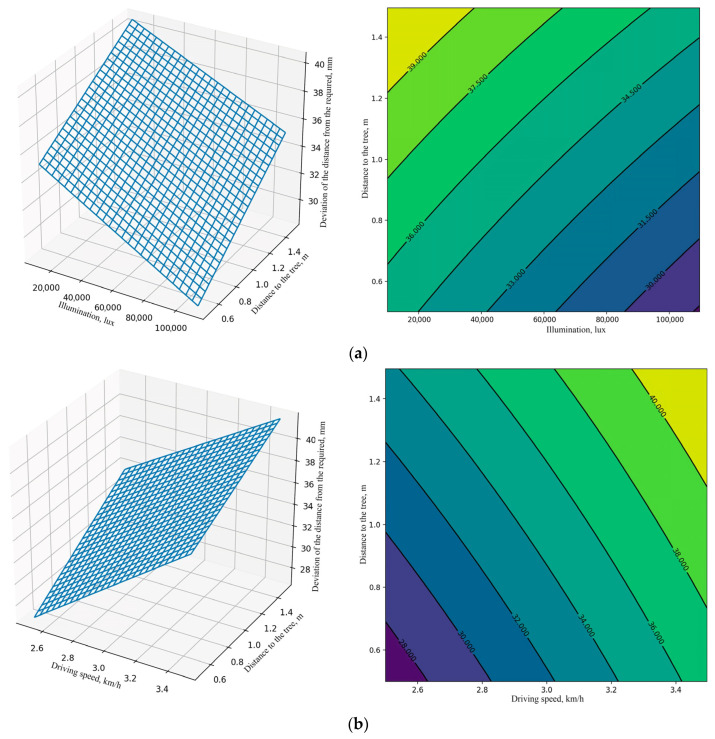

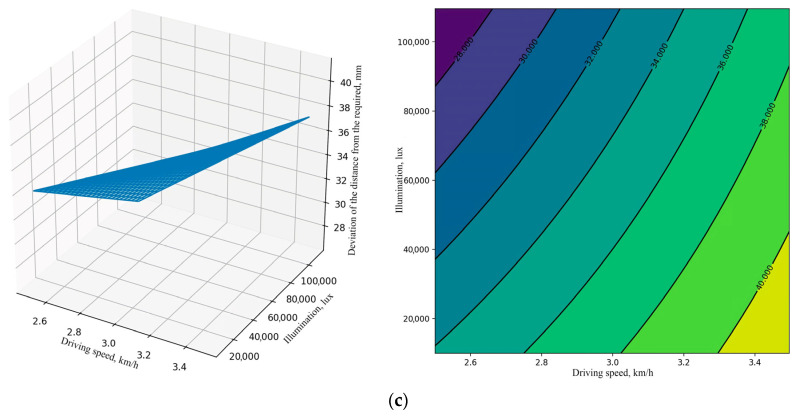
Graphs of the projections of three-dimensional response surfaces on planes according to the optimum: (**a**) y = f(x2, x3) at x1 = const; (**b**) y = f(x1, x3) at x2 = const; (**c**) y = f(x1, x2) at x3 = const.

It was established that the extremum of the response function of the mathematical model is in the range of changes in the factors. Graphical interpretations for a three-variable function and projection diagrams illustrating three-dimensional response surfaces onto a plane are shown in Figure A1 in Appendix A.

The maximum and minimum values of the response function and the corresponding coefficient values in coded and natural forms were determined (Table 4).

Thus, it was established that, to increase the accuracy of the movement of an autonomous vehicle along a given positioning trajectory using a LiDAR sensor, the most effective modes are as follows: travel speed: 2.5 km/h; illumination: 109,600 lux; distance to the tree: 0.5 m. At the same time, the minimum deviation from the given trajectory was 0.7 cm, and the average linear deviation was 2.8 cm.

The maximum deviation from the given trajectory under the worst conditions (speed: 3.5 km/h; distance to the tree: 1.5 m; and illumination: 10,000 lux) did not exceed 4.6 cm.

### 4.4. Planning and Results of Experiment in the Field

The field experiment was carried out in triplicate. The factorial design of the experiment and the planning matrix for various traffic modes and lighting conditions were similar to those of the laboratory experiment and are presented in Table 3. As in the laboratory experiment, the output parameter was the deviation in the distance to the tree from the desired value. Statistical processing of the obtained results of the full-scale experiment was carried out. The values of the input and output coefficients in coded and natural forms are given in Table 5.

As a result of the research, the linear interpolation was determined, and the variance in the reproducibility in parallel experiments was found. The results of the statistical processing of the data from the factorial experiment are presented in Table 6.

The coefficients of the mathematical model were found. The equation of the mathematical model was obtained in coded (7) and natural (8) forms:

y = 58.581 + 6.249 · x1 + (−3.912) · x2 + 3.249 · x3 + 0.916 · x1 · x2 + 0.251 · x1 · x3 + 0.416 · x2 · x3 + (−2.416) · x1 · x2 · x3(7)

y = 64.696 + (−2.305) · x1 + (−32.311) · x3 + 12.603 · x1 · x3 + (−0.0002) · x1 · x2 · x3(8)

The analysis of the mathematical model according to the Fisher criterion made it possible to determine the adequacy of the model ((F_p_(1.23) < F_t_(4.49)). As a result of the transformation, three variants of the mathematical model were obtained at y = f(x2, x3) at x1 = const (level 0; natural value: 3 km/h), y = f (x1, x3) at x_2_ = const (level 0 level; natural value: 60,000 lux) and y = f (x1, x2) with x3 = const (level 0; natural value: 1 m). Mathematical models taking into account the constant factor take the following forms:

y = 64.696 + (−6.915) · 3.0 + 5.499 · x2 + (−0.0006) · x1 · x2(9)

y = 17.60 + 11.495 · x1 + 3.489 · x2 + 1.005 · x1 · x2(10)

y = 32.386 + 10.298 · x1 + (−0.0002) · x1 · x2(11)

**Figure A2:**
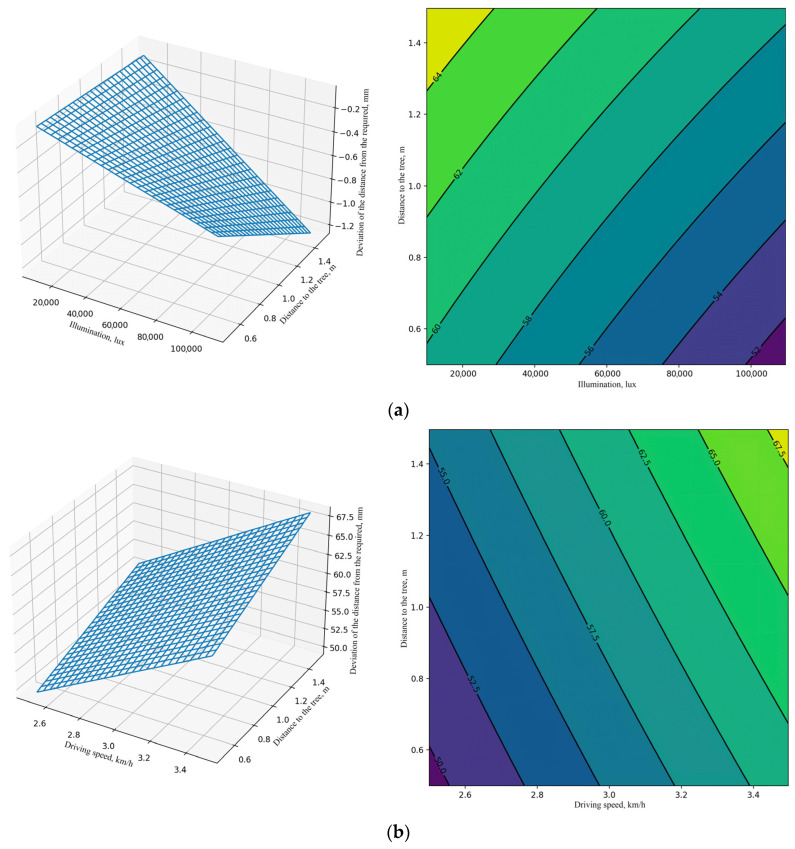

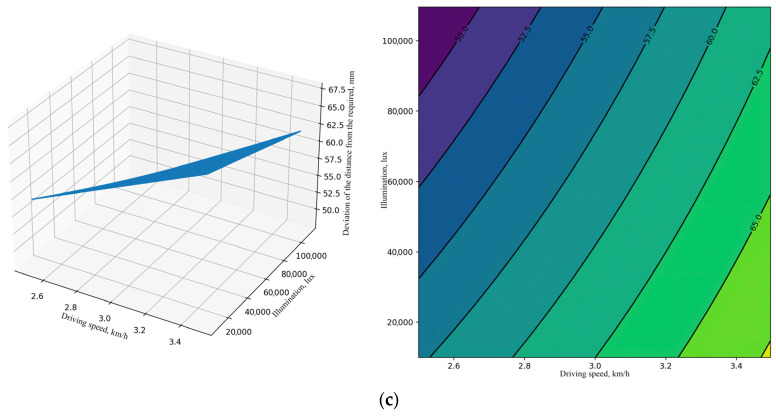
Projection graphs of three-dimensional response surfaces on planes according to the optimum: (**a**) y = f(x2, x3) at x1 = const; (**b**) y = f(x1, x3) at x2 = const; (**c**) y = f(x1, x2) at x3 = const.

It was established that the extremum of the response function of the mathematical model is within the range of the factor variation. Graphical interpretations of the function of three variables and projection diagrams of three-dimensional response surfaces on planes are shown in Figure A2 in Appendix A.

The maximum values of the response function and the corresponding optimal values of the coefficients in coded and natural forms were determined (Table 7).

## 5. Discussion

### 5.1. Problems Encountered during the Study and Ways to Solve Them

During the development of a motion control system for a robotic platform, several key issues arose that required careful analysis and effective solutions. The conditions in the field turned out to be more difficult than those in the laboratory, which reduced the positioning accuracy of the robotic platform. Additional calibration and tuning of the sensors were carried out, as well as the optimization of the data-processing algorithms to take into account the variable field conditions. The lighting conditions had a significant impact on the performance of the LiDAR sensor. An algorithm was used to compensate for changes in the lighting, incorporating a feedback mechanism to correct the route in real time and pre-adapted LiDAR settings to ensure a stable performance under different lighting conditions. The A* algorithm code was optimized to improve the performance of the hardware resources, and parallel computing was used to speed up the route-planning process. Working in the field involves interactions with various objects and people, requiring a high level of safety. An emergency braking system was used to avoid collisions, which worked based on obstacle detection. The large amount of data collected from a LiDAR sensor requires efficient storage and processing. The optimal data storage method was selected to ensure efficient operation. 

### 5.2. Comparison of Laboratory and Field Research Results

Field conditions, compared to laboratory conditions, present a number of unique factors that can significantly influence the positioning accuracy of robotic platforms. In field conditions, due to natural features, the soil surface may be uneven, which introduces significant uncertainty into the movement of the robotic platform and significantly reduces the positioning accuracy.

In this regard, it is necessary to use highly sensitive sensors and develop algorithms that can compensate for changes in the soil surface. Weather factors such as rain, snow or fog also affect LiDAR sensors, affecting their accuracy. Weather conditions highlight the importance of developing adaptation systems that can cope with changes in visibility and ensure a stable operation in different weather conditions. In the field, biological objects, such as plants, stones and various obstacles, can have a variety of shapes and sizes, unlike in the laboratory, where artificial plant models are used.

The algorithm must be able to effectively detect and adapt to a variety of biological objects and their size parameters. Laboratory conditions are more controlled than field conditions, making the positioning task easier. In real-life conditions, factors such as the presence of people, animals or various objects may change dynamically in an unpredictable scenario.

Statistical analysis of the experiment results showed that to improve the accuracy of an autonomous vehicle moving along a given trajectory using a LiDAR sensor under field conditions, the following modes are the most effective: travel speed: 2.5 km/h; illumination: 109,600 lux; distance to the tree: 0.5 m. At the same time, the minimum deviation from the given trajectory was 3.9 cm, and the average linear deviation was 6.15 cm.

Under the worst conditions (speed: 3.5 km/h; distance to the tree: 1.5 m; illumination: 10,000 lux), the maximum deviation from the given trajectory is no more than 8.4 cm. Mathematical models obtained under laboratory and field conditions have different coefficients and structures. The results show that real field conditions can differ significantly from laboratory ones, which makes more thorough testing and optimization necessary for the stable and accurate operation of the robotic platform in various operating scenarios. Under field conditions, the coefficients for each of the factors are significantly higher compared to those under laboratory conditions. This indicates a stronger influence of each of the factors (speed of movement, illumination and distance to the tree) on the deviation value in real conditions.

An analysis of the graphs obtained based on the results of the laboratory [51] and field studies showed that deviations from a given trajectory in the field turned out to be higher than in the laboratory. The calculation of the relative change for each characteristic obtained from the results of the laboratory and field studies showed that, as a percentage, the maximum deviation from a given trajectory in the field increased by 82.61%, the minimum deviation increased by 457.14% and the average linear deviation increased by 119.64%, compared with the laboratory conditions. A comparison of the coefficients of variation for each characteristic between the laboratory and field conditions showed that, at the maximum deviation from the given trajectory, the value of the coefficient of variation under field conditions (28.57%) is higher than that under laboratory conditions (26.09%). This may indicate a higher variability in the results in the field.

For the minimum deviation from the given trajectory, the value of the coefficient of variation in the field (85.71%) is significantly higher than that in the laboratory (46.15%). This may indicate a lower degree of variability in the results under laboratory conditions. For the average linear deviation, the value of the coefficient of variation in the field (32.14%) is higher than in the laboratory (19.51%). This may also indicate a higher variability in the results in the field. A high value of the coefficient of variation may indicate more unstable results and a greater influence of external factors in the field. This may be due to differences in real conditions, which may not always be accurately reproduced in the laboratory. Factors such as surface irregularities, non-uniform lighting and other external influences affect the operation of the LiDAR and the accuracy of the movement of the robotic platform. An analysis of the results of the study showed the possibility of using a robotic platform and LiDAR sensor to perform various technological operations in the field. They can be effectively applied for mobile autonomous navigation in various scenarios with positional movement when performing operations such as harvesting, spraying, processing near-trunks and monitoring.

In the articles [54,55,56,57,58,59,60], the authors conducted studies similar to ours. The Portuguese researchers D. Gomes et al. proposed automatic shape and position detection using a two-dimensional (2D) industrial laser to extract three-dimensional (3D) data, where object motion adds a third dimension through the laser beam [54]. The Indian researchers S. Vadapalli et al. proposed the 3D trajectory control of an autonomous underwater robotic vehicle using a backstepping approach based on robust feedback [55]. An article by the Chinese scientists R. Wang et al. [56] presents a proposed trajectory-tracking and obstacle-avoidance-planning algorithm for robotic fish using nonlinear model predictive control (NMPC).

In the study [57], the authors from Greece conducted the closest research to ours for a rail robot in a greenhouse—they built a route trajectory and visualized the movement using a LiDAR sensor. In this case, the mathematical apparatus of finite-state machines was used, as well as neural networks (YOLO v.8). The navigation route of a tracked vehicle in a pear orchard using a point cloud combined with precise positioning constructed using LiDAR was performed by Chinese researchers in the paper [58]. The authors claim that this combination provides the most accurate navigation and mapping, while moving away from the GNSS. At the same time, Brazilian researchers in [59] propose a combined navigation method based on data obtained from LiDAR, the GNSS and an RGB camera. The authors substantiated the effectiveness of various methods at different stages of route construction depending on the obstacles and the visibility of each type of signal. The authors of [60] propose a new trajectory generation method for autonomous-excavator training and planning applications. The method transforms an arbitrary slow and intermittent excavation trajectory and optimizes the trajectories in time and in the jerk aspect. A spline is used to connect these waypoints, which are topologically equivalent to the human learning path.

In our subsequent research on navigation, we also plan to use convolutional neural networks to refine the route. We used this toolkit to identify an apple when it was picked from a tree [16,17].

### 5.3. Limitations of or Potential Areas for Improvement in Sensor Applications

The VLP-16 LiDAR sensor, while effective, also has some limitations and areas for potential improvement, including the number of beams. This reduces the resolution and accuracy in some use cases. To improve the detail of the perception of the environment, it is necessary to use models with higher resolutions. The limited field of view of the VLP-16 LiDAR makes it difficult to fully cover the environment. Wide-angle models or multiple LiDAR sensors are required to achieve full coverage. Adverse weather conditions have an impact on the performance of the LiDAR VLP-16, especially in rainy conditions. Research on and the implementation of methods for compensating for the impact of weather factors on the sensor operation will improve the positioning accuracy of robotic platforms.

VLP-16 may have difficulty detecting small or distant objects. Work on detection algorithms that can effectively recognize and classify various objects will also improve the positioning accuracy. Limited energy resources may be problematic for some mobile platforms. Work on optimizing the power consumption will allow the LiDAR sensor to be used more efficiently in different scenarios for a wider range of applications.

### 5.4. Prospects for Future Research

There are several promising areas for future research on and improvements in the motion control of robotic platforms using the VLP-16 LiDAR sensor:

- The integration of more advanced machine learning methods that do not require large computing resources, which will allow the system to adapt to a variety of agricultural conditions and develop decision-making algorithms that can learn from experience;
- Research on and the integration of additional sensors and technologies, such as cameras, radars and augmented reality, to create a more complete and reliable perception and navigation system;
- The development of more effective algorithms for detecting and classifying obstacles, taking into account the diversity of biological objects and their shapes and sizes, as well as changes in lighting, weather conditions and soil surface types;
- The development of energy-efficient propulsion and energy management strategies to extend the autonomy of robotic platforms;
- The collaborative control of multiple robotic platforms to improve efficiency and coordination in agricultural operations.

These lines of research could lead to significant improvements in automation and robotics in agriculture, optimizing processes and increasing productivity while minimizing the environmental impact.

## 6. Conclusions

As a result of the research, a motion control system for the robotic platform was developed based on laser ranging, the A* algorithm for traversing the graph and the identification of the optimal path. To find the optimal navigation graph in an artificial horticultural environment, an application was developed using the C# programming language and Visual Studio 2019.

It was substantiated that the use of the A* algorithm with the Velodyne Puck VLP-16 LiDAR sensor allows for finding the shortest path from the starting point of the trajectory to the given intermediate and end points, analyzing all the trajectory options step by step. The Rviz visualization environment implements the possibility of designing a route for the movement of a robotic platform, building a map by scanning a previously unknown surrounding space using a LiDAR sensor and updating the resulting map at each step of the algorithm in real time.

The modular architecture of the developed motion control system for the robotic platform allows it to be supplemented with the following extensions:

- A motion control system using a scenario for following a person using a technical vision system and neural network;
- A system for preventing collisions with people, animals and obstacles based on ultrasonic sensors;
- A traffic control system based on inertial and satellite navigation and the calculation of the path to be overcome.

An analysis of the operating parameters of the LiDAR sensor showed that it provides a high degree of positioning accuracy under various lighting conditions at different speeds in the aisles of an artificial horticulture model 3 m wide with an inter-stem distance of 1.5 m and a tree crown width of 0.5 m. The use of existing modern sensors with an optical rangefinder with a resolution of 4.5 million pixels, a frame rate of 25 FPS and the ability to automatically adapt to the level of illumination in combination with stereo cameras and GPS/GLONASS navigation will further improve the accuracy and ensure the autonomous performance of the units of the main technological operations in intensive horticulture with a deviation from a given trajectory of no more than 1.5–2 cm, which satisfies the agrotechnical requirements.

## Figures and Tables

**Table 1:** Results of pathfinding in a graph with obstacles using the four algorithms in the Github PathFindings web service.

Criteria	Algorithm A*	Breadth-First Search Algorithm	Best-First Search Algorithm (Search for the First Best Match)	Dijkstra’s Algorithm
Length, m	327.81	327.81	331.95	327.81
Time, min	7.10	3.50	4.80	5.70
Number of operations, units	2118.00	2211.00	1930.00	2211.00
**Color designations**	Worst indicator	Best indicator	Good indicator	Bad indicator

**Table 2:** Equipment for conducting experiments under laboratory conditions.

No.	Parameter	Type	Value
1	Light-level measurement	Spectrometer-impulse meter	Uprtek MF250N
2	Light source	Fluorescent gas-discharge lamps	Super Lamp Holder SLH3 45 W 220 v 5500 K RoHS
3	Analysis of accuracy of movement along a given trajectory	Rangefinder	Benewake TFmini Plus
4	Control and storage of experimental data	Program	Advanced Serial Port Monitor 3.5.3
5	Distance between the robotic platform and the artificial model	LCD display	LCD Display MT-20S4A-I
6	Data processing	Computer	Intel NUC

**Table 3:** Time-complexity comparison.

	Breadth-First Search Algorithm ($O(\left|V\right|+\left|E\right|)$)	Algorithm A* $O(\left(\left|V\right|+\left|E\right|\right)log\;(|V|)$
Number of passed nodes, V	77	10
Number of fins, E	423	9
Time complexity	500	19

**Table 4:** Results of statistical processing of data from a field experiment in an artificial garden.

Extremum of Response Function	Speed of Movement, km/h	Illumination, lx	Distance to Tree, m
Y_опт._ = 28.362;	x1 = 0 (3)	x2 = 109,600	x3 = 0.5
Y_опт._ = 26.67;	x1 = 2.5	x2 = 0 (60,000)	x3 = 0.5
Y_опт._ = 26.2;	x1 = 2.5	x2 = 109,600	x3 = 0 (1);

**Table 5:** Experiment planning matrix in the field.

Experience Number	Planning Matrix, Values of Variable Factors			Experimental Results, Output-Factor Values		
	x1, Speed of Movement, km/h	x2, Illumination, lx	x3, Distance to Tree, m	y1	y2	y3
1	2.5	110,000	0.5	41	42	42
2	3.5	10,000	0.5	63	62	62
3	2.5	10,000	1.5	57	58	57
4	3.5	110,000	1.5	63	63	64
5	2.5	10,000	0.5	56	57	58
6	3.5	110,000	0.5	59	61	61
7	2.5	110,000	1.5	53	54	53
8	3.5	10,000	1.5	73	74	73

**Table 6:** Results of statistical processing of experimental data under laboratory and field conditions.

Indicator	Value	
	Laboratory Experiments	Field Experiments
$\mathrm{Total}\;\mathrm{variance},\;\sigma^{2}$	4.33	7.33
$\mathrm{Maximum}\;\mathrm{variance},\;\sigma_{max}^{2}$	1.33	2.33
$\mathrm{Cochran}’s\;\mathrm{design}\;\mathrm{criterion},\;G_{pac}$	0.30	0.31
$\mathrm{Cochran}’s\;\mathrm{tabular}\;\mathrm{test},\;G_{Taбл}$	0.51	0.51
Homogeneity of dispersions	Homogeneous	Homogeneous
$\mathrm{Adequacy}\;\mathrm{dispersion},\;\sigma_{a}^{2}$	0.50	1.13
$\mathrm{Reproducibility}\;\mathrm{dispersion},\;\sigma_{B}^{2}$	0.54	0.91
$\mathrm{Fisher}’s\;\mathrm{design}\;\mathrm{criterion},\;F_{p}$	0.93	1.23
$\mathrm{Tabular}\;\mathrm{value}\;\mathrm{of}\;\mathrm{the}\;\mathrm{Fisher}\;\mathrm{criterion},\;F_{T}$	$\mathrm{Undefined},\;\sigma_{a}^{2}<\sigma_{B}^{2}$	4.49
Adequacy of the mathematical model	The model is adequate ($F_{p}<F_{T}$)	The model is adequate ($F_{p}<F_{T}$)

**Table 7:** Results of statistical processing of field experiment data.

Extremum of Response Function	Travel Speed, km/h	Illumination, lx	Distance from Tree, m
Y_опт._ = 51.035;	x1 = 0 (3)	x2 = 109,600	x3 = 0.5
Y_опт._ = 49.335;	x1 = 2.5	x2 = 0 (60,000)	x3 = 0.5
Y_опт._ = 47.539;	x1 = 2.5	x2 = 109,600	x3 = 0 (1);

## Data Availability

Data are contained within the article.
